# The Environment Affects Epistatic Interactions to Alter the Topology of an Empirical Fitness Landscape

**DOI:** 10.1371/journal.pgen.1003426

**Published:** 2013-04-04

**Authors:** Kenneth M. Flynn, Tim F. Cooper, Francisco B-G. Moore, Vaughn S. Cooper

**Affiliations:** 1 Department of Molecular, Cellular, and Biomedical Sciences, University of New Hampshire, Durham, New Hampshire, United States of America; 2 Department of Biology and Biochemistry, University of Houston, Houston, Texas, United States of America; 3 Integrated Bioscience Program, University of Akron, Akron, Ohio, United States of America; Washington University School of Medicine, United States of America

## Abstract

The fitness effect of mutations can be influenced by their interactions with the environment, other mutations, or both. Previously, we constructed 32 ( = 2^5^) genotypes that comprise all possible combinations of the first five beneficial mutations to fix in a laboratory-evolved population of *Escherichia coli*. We found that (i) all five mutations were beneficial for the background on which they occurred; (ii) interactions between mutations drove a diminishing returns type epistasis, whereby epistasis became increasingly antagonistic as the expected fitness of a genotype increased; and (iii) the adaptive landscape revealed by the mutation combinations was smooth, having a single global fitness peak. Here we examine how the environment influences epistasis by determining the interactions between the same mutations in two alternative environments, selected from among 1,920 screened environments, that produced the largest increase or decrease in fitness of the most derived genotype. Some general features of the interactions were consistent: mutations tended to remain beneficial and the overall pattern of epistasis was of diminishing returns. Other features depended on the environment; in particular, several mutations were deleterious when added to specific genotypes, indicating the presence of antagonistic interactions that were absent in the original selection environment. Antagonism was not caused by consistent pleiotropic effects of individual mutations but rather by changing interactions between mutations. Our results demonstrate that understanding adaptation in changing environments will require consideration of the combined effect of epistasis and pleiotropy across environments.

## Author Summary

The fitness effect of beneficial mutations can depend on how they interact with their genetic and external environment. The form of these interactions is important because it can alter adaptive outcomes, selecting for or against certain combinations of beneficial mutations. Here, we examine how interactions between beneficial mutations favored during adaptation of a lab strain of *Escherichia coli* to one simple environment are altered when the strain is grown in two novel environments. We found that fitness effects were greatly influenced by both the genetic and external environments. In several instances a change in environment reversed the effect of a mutation from beneficial to deleterious or caused combinations of beneficial mutations to become deleterious. Our results suggest that a complex or fluctuating environment may favor combinations of mutations whose interactions may be less sensitive to external conditions.

## Introduction

The extent to which mutations interact with their genetic background (epistasis) and the role such interactions play in evolution is not well understood [1], [2]. Initial expectations were that epistatic interactions, defined as non-additive interactions among mutations, were common, causing fitness landscapes to be rugged and limiting the number of selectively accessible mutational paths [3]. Although early work revealed few such interactions (reviewed in [4]), more recent studies of defined combinations of mutations have revealed abundant epistasis in a range of systems [5]–[17]. Studies that focused on interactions among beneficial mutations have often found a tendency for antagonism [18]–[21], which is consistent with epistasis having a predictable influence on the curvature of fitness peaks in constant environments [22], [23].

In addition to interactions within a genome, mutations can also interact with the external environment [24]–[29]. Moreover, phenotypic plasticity and epistasis can combine so that the fate of a mutation depends on both the environment and its genetic background [27], [30]. This kind of dependence can have important evolutionary consequences. For example, Wright considered how fluctuating conditions — most often population size, but also the external environment — could change the sign of epistatic interactions and allow populations to evolve along otherwise maladaptive paths [31]–[35].

Relatively few studies have examined how epistasis and plasticity combine to influence mutational effects. One study that did found that all of 18 transposon insertion mutations were affected by either epistasis or plasticity, with half being affected by both [28]. It remains unclear, however, how interactions between beneficial mutations, which might be expected to depend strongly on a particular selective environment, will be affected by changes in the external environment. Such an understanding is vital for addressing questions concerning the course of adaptation in fluctuating conditions. For example: can the magnitude and sign of epistasis change with the external environment? If so, are there any overarching features of mutation interactions, e.g., a tendency towards antagonism, that nevertheless remain consistent? A few studies have begun to address these questions by examining how epistasis between pairs of mutations changes with genomic [36], [37] and environmental [38] contexts.

Here, we expand this investigation of how the external environment affects epistatic interactions between five beneficial mutations that fixed in one population of a long-term *Escherichia coli* evolution experiment [19]. We first screened the response of the ancestor and the evolved genotype having all five mutations over a total of 1,920 external environments. Next, we measured the fitness of a set of 32 ( = 2^5^) strains comprising all mutation combinations in the two environments with the most extreme opposing plasticity. These measurements allowed us to isolate effects of epistasis (GxG), interactions between mutations and the environment (GxE), and interactions between epistasis and the environment (GxGxE) on the fitness of defined genotypes. More generally, we investigated how the adaptive landscape, and the indirect consequences of each mutational step, might change with the external environment.

## Results

### Interactions between beneficial mutations and their external environment

To determine how phenotypic plasticity changes following an adaptive walk, we used Biolog plates to compare the respiration (a measure of catabolic activity) of the ancestor and the strain containing five beneficial mutations (hereafter, rtsgp, where each letter indicates a mutation in a gene or gene region as follows: r = *rbs*, t = *topA*, s = *spoT*, g = *glmUS* and p = *pykF*) in 1,920 different environments. On average, rtsgp exhibited enhanced respiration over the ancestor (mean = 84.37±1.39 (SEM) compared to 72.45±1.14 (SEM); paired *t*-test, *t_1919_* = 26.28, *P* = <0.0001) with significant differences in 203 environments (see Materials and Methods for criteria), involving 171 gains of function and 32 losses of function (Table S1). These environments contained 28 alternative carbon sources, 34 alternative nitrogen sources, five alternative phosphate sources, ten nutritional supplements, and 126 “stressors” including antibiotics and other potentially toxic chemicals. Providing a useful control, one of the carbon sources in which the rtsgp strain had decreased respiration was D-ribose, which was expected due to a large deletion of the *rbs* operon in this strain [39]. We confirmed that measured respiration changes reflected growth rate changes in eight of the environments (six gains of function and two losses of function) by direct growth comparisons (Table S2).

We focused on two environments that revealed large differences in respiration between rtsgp and the ancestor to examine how genotype and environment interact to affect fitness. The largest relative increase in respiration was in the presence of EGTA, a Ca^++^/Mg^++^chelator [40]. The largest decrease was in the presence of guanazole, a ribonucleotide DP reductase inhibitor [41]. In direct fitness competitions comparing rtsgp to its ancestor, rtsgp was significantly more fit in the environment containing the original selection medium (DM25, a minimal salts medium supplemented with glucose) supplemented with EGTA than in the environment containing the selection medium alone (DM25+EGTA: fitness = 1.497±0.068 (95% CI); DM25: 1.299±0.061 (95% CI), *t_9_* = 4.973, *P* = 0.0008). By contrast, rtsgp was less fit in DM25 supplemented with guanazole than in the selection environment (DM25+guanazole: fitness of 1.116±0.029 (95% CI); DM25: 1.299±0.061 (95% CI), *t_9_* = −5.779, *P* = 0.0003).

**Figure 1:**
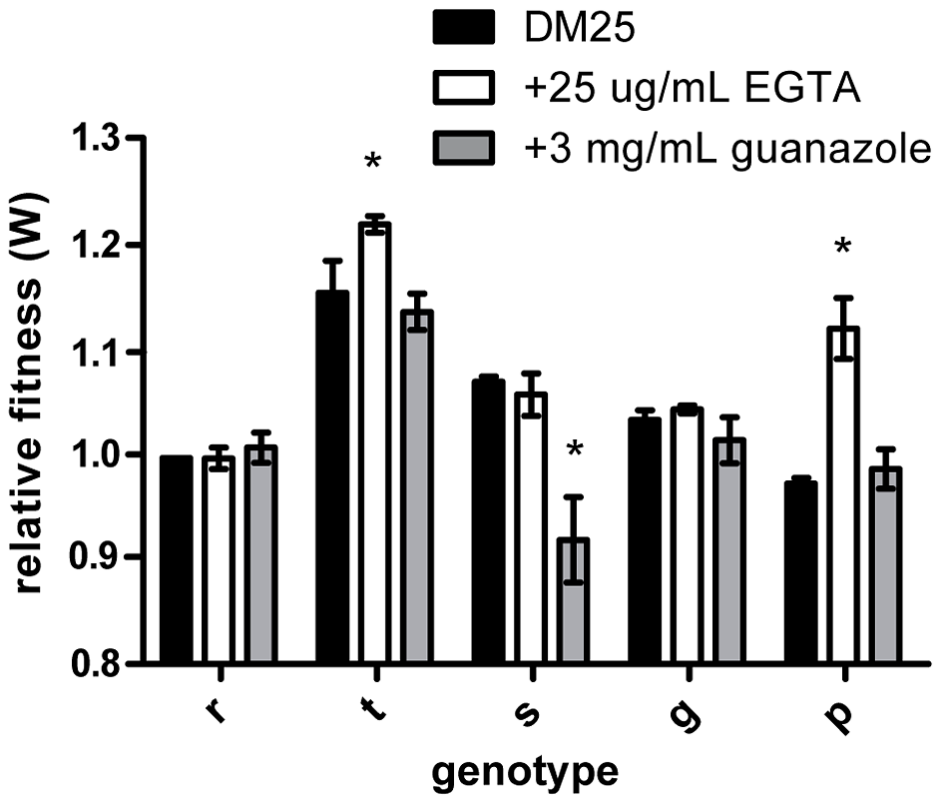
Effects of beneficial mutations in alternative environments. Genotypes are designated as single letters and define alleles: *rbs* (r), *topA* (t), *spot* (s), *glmS* (g), and *pykF* (p). Fill color defines the environment: black, DM25, white, EGTA, and grey, guanazole. Mutational effects were determined to depend on the environment using an ANOVA. Asterisks represent significance based on a *P* value < 0.05.

To examine the underlying genetic basis of this phenotypic plasticity, we quantified the fitness effect of each individual mutation in all three environments. The relative fitness of three of the five mutations significantly depended on the external environment (*rbs*: *F_2,11_* = 1.100, *P* = 0.367; *topA*: *F_2,8_* = 15.506, *P* = 0.002; *spoT*: *F_2,10_* = 31.389, *P*<0.0001; *glmUS*: *F_2,10_* = 3.513, *P* = 0.070; *pykF*: *F_2,10_* = 149.730, *P*<0.0001) (Figure 1). In summary, three of the individual mutations present in the rtsgp genotype produced effects that differed significantly across environments.

### Fitness varies across external environments due to plasticity and epistasis

The above results demonstrate that the effect of individual mutations depend on the environment (i.e., G×E). However, it is also possible that interactions between mutations depend on the environment (i.e., G×G×E), which would further influence the topology of the fitness landscape and make it much more difficult to predict the influence of environmental changes on evolutionary outcomes. To examine G×G×E we measured the fitness of all combinations of the five beneficial mutations in the two focal environments (i.e., the selection environment supplemented with EGTA and guanazole).

**Figure 2:**
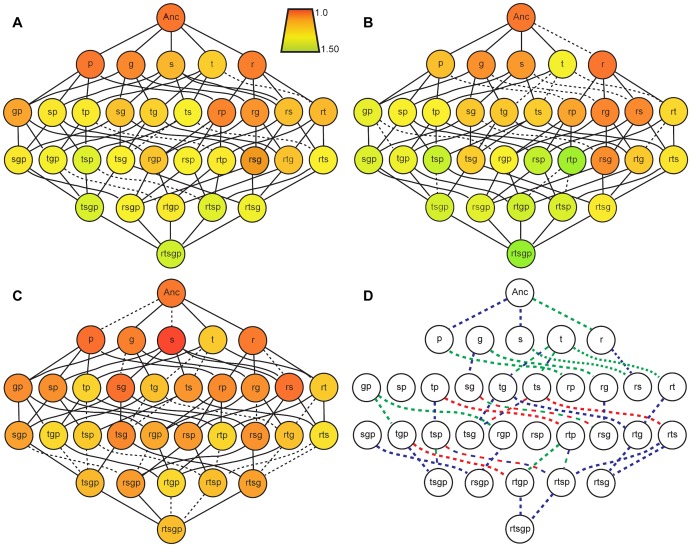
Fitness landscapes in the selection environment and in two novel environments. Genotypes are designated as single letters as defined previously. A, B, C: Node color indicates the fitness of a genotype relative to the ancestor in that environment. Solid lines indicate a selectively accessible step that increases genotype fitness and dashed lines indicate steps corresponding to fitness decreases. D: Colored dotted lines indicate selectively inaccessible mutational paths with a neutral or deleterious effects on fitness given mean relative fitness values (Tables S5 and S6); line color defines the environment: red, DM25; green, EGTA; blue, guanazole. DM25 data is adapted from Khan et al [19].

Fitness of each of the 32 genotypes comprising each mutation combination was quantified in both novel environments and compared with prior findings in the original selection environment [19] (Figure 2). To get some overall indication of the influence of environment on mutation effects we compare the number of “selectively accessible” mutational paths connecting the ancestor and rtsgp [42]. Although a different set of beneficial mutations would presumably be followed in guanazole and EGTA environments, considering a common set of genotypes allows a direct comparison of the effect of environment in altering selection pressures as a result of GxE and GxGxE. Of the 120 ( = 5!) paths connecting the ancestor and rtsgp, 86 had monotonically increasing fitness in the selection environment [19]. By contrast, only 43 paths in EGTA and 2 paths in guanazole are selectively accessible (Figure 2, Tables S3, S4). (The small number of selectively accessible paths in guanazole reflects, in large part, that rts and not rtsgp was the most fit genotype (Table S6).) In all, nine mutational steps became significantly deleterious, six in the EGTA environment and three in the guanazole environment (Tables S3 and S4), although only three of these steps in the EGTA environment remain significantly deleterious when we correct for multiple comparisons. Nevertheless, differences in the number of selectively accessible paths available in different environments clearly indicate that environment affects landscape topology and selective constraints.

**Figure 3:**
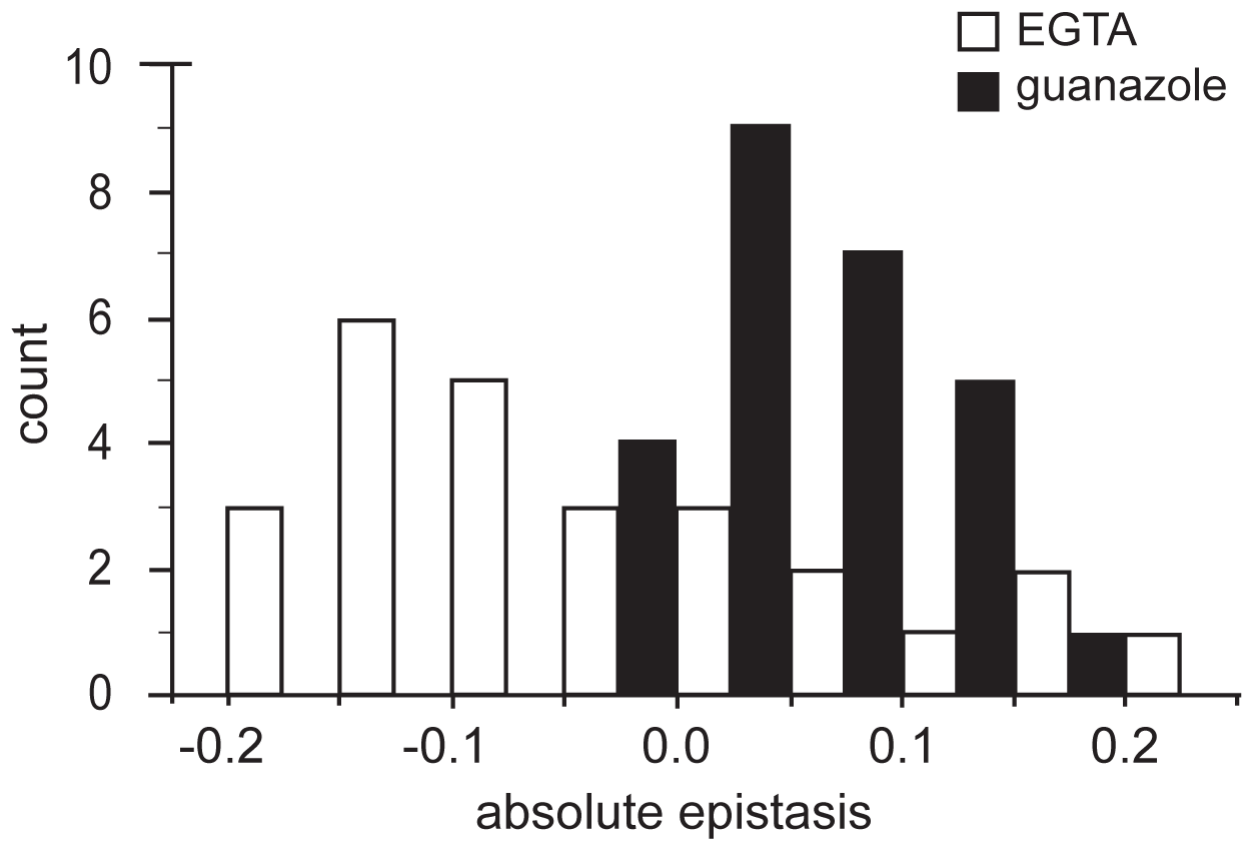
Distributions of epistatic effects in two environments. Observed and expected fitness were compared for 26 genotypes containing two or more mutations in two environments. Absolute epistasis was calculated as described in the text.

To further examine the patterns of epistasis in the novel environments we focused on the effect of epistasis in determining the fitness of individual genotypes (Tables S5, S6). In the EGTA environment mean epistasis was slightly, but not significantly, negative (mean absolute epistatic deviation, **ε_m_** = −0.039±0.046 (95% C.I.), *t_25_* = −1.740, *P* = 0.094) (Figure 3). In the guanazole environment mean epistasis was significantly positive (**ε_m_** = 0.057±0.022 (95% C.I.), *t_25_* = 5.303, *P*<0.0001) (Figure 3). In total, 16 and 5 genotypes exhibited significant epistasis in EGTA and guanazole, respectively (Tables S4, S5). Both environments also displayed markedly different effects of higher-order epistasis involving interactions between at least three mutations. In the EGTA environment, genotypes tended to be more fit than expected from the sum of the relevant lower-order interactions (mean higher-order epistatic deviation = 0.229±0.191 (95% C.I.), *t_15_* = 2.556, *P* = 0.022). The opposite effect was seen in the guanazole environment (mean higher-order deviation = −0.247±0.196 (95% C.I.), *t_15_* = 2.693, *P* = 0.017).

**Figure 4:**
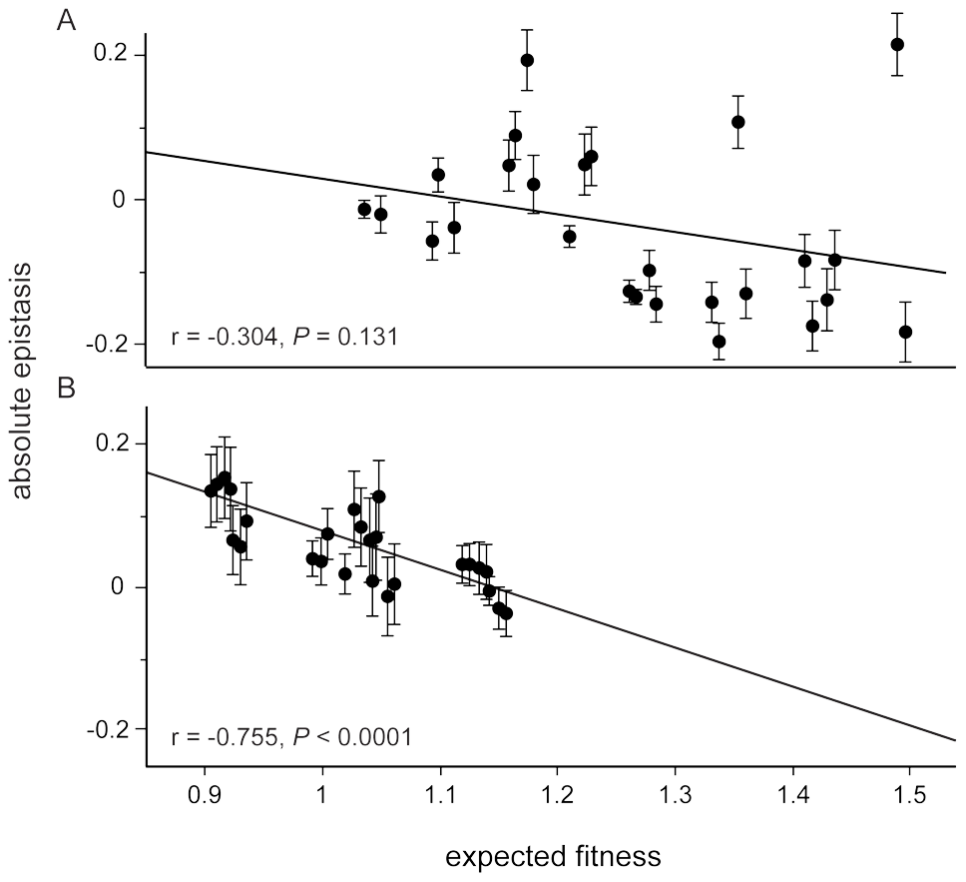
Relationship between relative epistasis and expected fitness assuming no epistasis in each foreign environment. Each point refers to one of the 32 genotypes assayed for fitness in both environments (a, EGTA, b, guanazole). Error bars represent the standard deviation approximated through the method of error propagation. The solid lines are the best linear fit with the text below reporting the correlation (*r*) and significance (*P* values).

Considering only the mean effect of epistasis can miss other underlying patterns. For example, we previously found that the strength of negative epistasis between the five beneficial mutations increased with the expected fitness of the genotype in the selection environment, despite a lack of any mean effect [19]. This pattern has been reported in several other studies [18], [20], [21] and is consistent with interactions between beneficial mutations acting to slow the rate of adaptation. In the guanazole environment we found the same negative correlation between epistasis and expected fitness that was seen in the selection environment (*r* = −0.748) (Figure 4, Figure S1, Table S5 and S6). The same correlation was only weakly negative in the EGTA environment (*r* = −0.281) (Figure 4 and Figure S2). We also evaluated whether interactions between mutations and the environment, either EGTA or guanazole, contributed significantly to the overall variation in fitness, and found significant interactions between mutations (GxG), mutations and the environment (GxE), and interactions of both (GxGxE) (overall model: *F_63,261_* = 58.439, *P*<0.0001, Table S7, see Materials and Methods). Using variance partitioning, we determined that GxGxE interactions explained approximately 8% of the variance in fitness observed in our complete data set (Table S8).

Correlations between epistasis and expected fitness could reflect a general trend but could also be leveraged by outlying fitness or epistatic effects of an individual mutation. To distinguish between these possibilities we performed a series of ANCOVA analyses to test whether the presence or absence of each focal mutation influenced the overall relationship between epistasis and expected fitness (Figure S3 and S4). Only the *pykF* mutation explained a significant portion of the variation in the relationship between epistasis and expected fitness in the guanazole environment (Figure S3). Genotypes with this mutation tended to be more fit while the negative correlation, consistent with diminishing returns epistasis, with or without this mutation was retained.

In the EGTA environment, considering genotypes distinguished by the presence or absence of either *topA* or *pykF* mutations revealed their significant contributions to the overall pattern of epistasis (Figure S4). The *topA* mutation tended to effect epistasis so as to decrease fitness (mean epistasis of genotypes with *topA* = −0.088 compared to mean epistasis of genotypes without  =  0.042, *t_24_ = *3.272, *P* = 0.003) whereas *pykF* altered epistasis to generally increase genotype fitness (mean epistasis of genotypes with *pykF* = 0.007 compared to mean epistasis of genotypes without = −0.087, *t_24_ = *−2.156, *P* = 0.041). Genotypes lacking the *rbs* mutation again displayed a strong negative correlation between epistasis and expected fitness (*r* = −0.783), but adding the *rbs* mutation weakened the negative association between epistasis and expected genotype fitness without changing mean epistasis among these genotypes (Figure S4, genotypes with *rbs, r* = 0.089, compared to without *P* = 0.033; mean epistasis with *rbs* = 0.0001 compared to mean epistasis of genotypes without = −0.078, *t_24_ = *−1.720, *P* = 0.098).

### Epistasis is common in a variety of external environments

**Figure 5:**
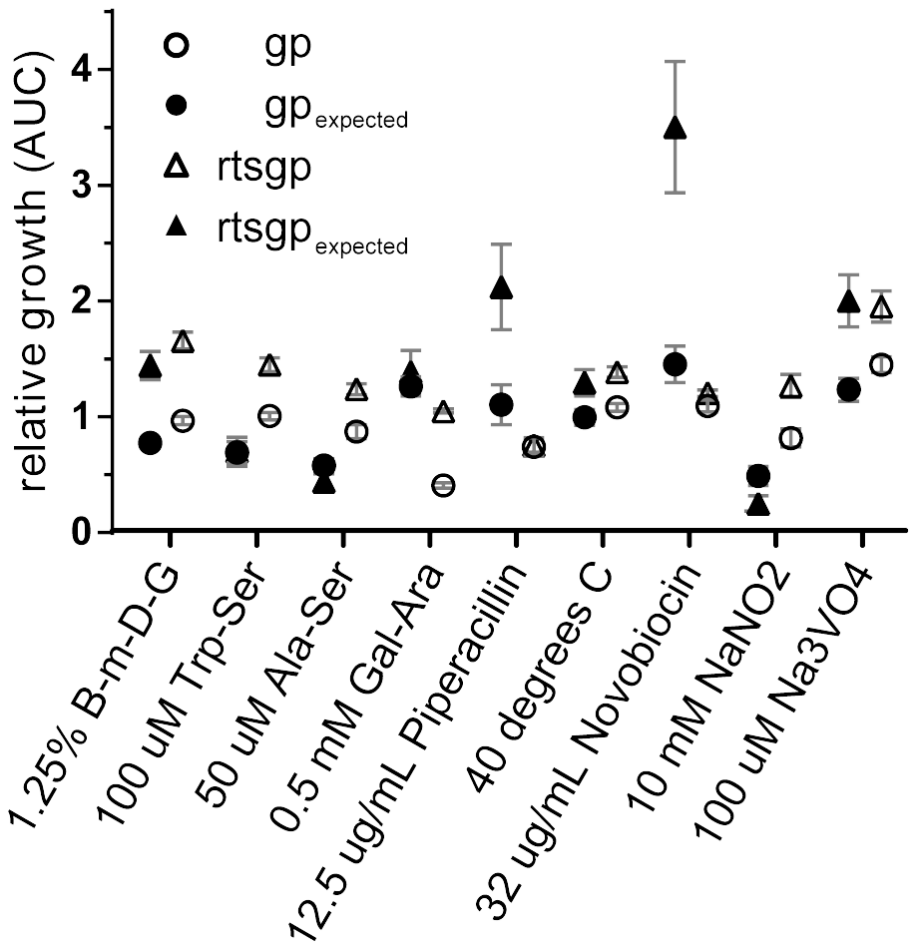
The magnitude and direction of epistatic effects on growth vary with external environment. Symbols represent growth (AUC) of a particular genotype relative to the ancestor (triangles = gp, circles = rtsgp). Filled symbols represent expected relative growth and open symbols represent observed relative growth based on a multiplicative model assuming no epistatic interactions. Differences between observed and expected values were determined using the t statistic (Table S9 and S10).

We used a higher-throughput approach using overall population growth (AUC, see Materials and Methods) as a proxy for fitness to assay for epistasis in nine additional environments. Seven of these environments were not expected to interact with the five mutations based on the initial Biolog screen comparing the rtsgp and ancestral strains (Table S1, Materials and Methods). In each environment, the growth of each single mutant was compared with the ancestor, rtsgp, and a randomly selected double-mutant, gp (Figure 5). In seven of nine environments, growth of either rtsgp or gp differed significantly from additive expectations assuming no epistasis (Figure 5, Tables S9, S10). The nature of these interactions also changed with the environment. For example, gp was significantly less fit than expected in two environments and significantly more fit than expected in four environments (Table S9). In summary, the sign and magnitude of epistasis among generally beneficial mutations may vary widely even with relatively small changes in the external environment.

## Discussion

Recent theoretical work has applied population genetic models to empirically constructed fitness landscapes to make basic predictions about the likelihood of particular evolutionary outcomes [8], [14]. These outcomes depend crucially on the shape of the fitness landscape, which is determined by the form and extent of epistatic interactions between mutations. How sensitive these interactions, and therefore the repeatability of evolutionary outcomes, are to environmental change remains uncertain. To address this point experimentally we analyzed a set of strains including all combinations of the first five beneficial mutations that fixed during the adaptation of a population of *E. coli* to a constant laboratory environment (Table 1, [19]). By measuring the fitness of these strains in contrasting environments we generated two new empirical fitness landscapes that reveal how epistasis may change with the environmental context. Comparing these landscapes to the one determined in the original selection environment, we found interactions between mutations and their environment to be both common and complex.

**Table 1:** The five mutations in the order in that they arose and fixed in a population of *E. coli* from Lenski et al. 2002.

Order	Name	Gene	Mutation Type	Description	Fitness Gain*	Collective Gain^6^	Fitness Gain (EGTA)	Coll. Gain (EGTA)	Fitness Gain (guan)	Coll. Gain (guan)
**1**	**r**	***rbs***	IS150-mediated deletion of the rbs operon.	Loss of ribose catabolic function.	1.5%^1^	1.5%	0%	0%	0.5%	0.5%
**2**	**t**	***topA***	C-to-T non-synonymous substitution, H33Y.	Encodes topoisomerase I; this mutation increases DNA supercoiling.	13.3%^2^	15%	22%	16%	13%	14%
**3**	**s**	***spoT***	A-to-T non-synonymous substitution, K662I, leading to the change of a lysine into an isoleucine.	Metabolizes the signaling molecule ppGpp involved in the stringent response associated with starvation, resulting in global expression changes.	9.4%^3^	25%	6%	18%	−8%	18%
**4**	**g**	***glmS***	1 bp insertion in the BoxG1 region upstream of *glmUS*.	Involved in cell-wall biosynthesis resulting in larger cell-size.^4^	∼5%^5^	30%	4%	19%	1%	7%
**5**	**p**	***pykF***	Null mutation due to a premature stop caused by an insertion of IS150.	Enzyme involved in the Entner–Doudoroff pathway of central metabolism converting PEP→pyruvate. Also, a component of the Phosphotransferase system (PTS) involved in glucose uptake.	0%^6^	35%	12%	50%	−1%	12%

* Relative fitness in DM25, experimental evolution conditions, relative to the ancestor, REL606.

1 [39],

2 [62],

3 [65],

4 [66],

5 [61],

6 [57].

Previous work has shown that the diet breadth of 12 *E. coli* populations, including the population that was the source of the mutations used in our experiments, declined substantially during long-term evolution in a constant environment with a single carbon source [39], [43], [44]. However, it is difficult to distinguish if this trend was caused by few mutations of strong pleiotropic effect or if the beneficial substitutions display antagonistic pleiotropy in general. In an effort to distinguish these explanations, one study specifically focused on pleiotropic effects of beneficial mutations in five different environments. Mutations that were beneficial in the selected environment tended to be beneficial in others, and although there were exceptions, limited antagonistic pleiotropy was observed [45], [46]. Here, we also report limited antagonistic pleiotropy with five beneficial mutations with an increased sample size of 1,920 environments from our initial Biolog screen (Table S1). This result supports the inference that antagonistic effects may be limited to a subset of beneficial variation. Since both studies focused on a collection of beneficial mutations contributing to initial adaptation to a minimal glucose environment, we speculate that early adaptation may be characterized by niche expansion with limited cost [47].

Epistasis was frequent in all environments and generally followed a pattern of diminishing returns. Nevertheless, both the individual effects of mutations and their interactions were environmentally dependent, in several cases resulting in mutations changing from being beneficial to deleterious or neutral (Figure 1, Figure 4, Figure S2 and S3). Perhaps most strikingly, different numbers of paths to the rtsgp genotype were found in each environment, one of which featured a different global peak. Our results also suggest that selective constraints in fluctuating environments may depend on how the environment influences epistasis between contending adaptive alleles, and not just the pleiotropic effects of individual mutations alone (Table S5). For example, the *topA* and *glmS* mutations were more beneficial in the EGTA environment alone (positive pleiotropy) (Figure 1), but in combination the tg genotype was much less fit than expected (Table S4). Since the fitness of this genotype did not significantly deviate from expectations in the guanazole environment (Table S5), environmental effects on epistasis (GxGxE) were not predicted by GxE interactions. More broadly, these results indicate that variable fitness of a genotype under different conditions can arise from altered interactions among the alleles comprising that genotype and not from any single mutation. This conclusion is robust to fitness measurements in environments not found to effect rtsgp respiration, suggesting that it is not dependent on our initial focus on the two environments in which GxE was most extreme (Figure 5).

These interactions may be especially important in determining evolutionary outcomes given initially rugged fitness landscapes [14], [48], [49] or in naturally variable environments. In one study [17], a more beneficial allele was eventually outcompeted by a less fit allele because of epistatic limits to the adaptive path of the former allele. However, a different outcome may have occurred in a fluctuating or seasonal external environment. Given prevalent genotype-by-environment interactions, epistatic interactions producing low fitness intermediates could be alleviated in alternative environments and allow new combinations of alleles to overcome evolutionary dead-ends and rise to fixation. This process could represent a mechanism for maintaining conditional, yet beneficial, variation in the population [50]–[52]. As evidence, the three significantly maladaptive steps in the EGTA environment are alleviated by a shift to either the guanazole or the original selection environment (Figure 2). Fluctuating environments may therefore provide a solution to evolutionary dead-ends in an inherently rugged fitness landscape.

In summary, the combination of phenotypic plasticity and epistasis can strongly influence how an organism adapts to a new environment. Although the five mutations examined here would not likely be the same favored in these new environments, our results demonstrate that epistatic interactions are not static and can determine which trajectories are selectively accessible during an adaptive walk in a fluctuating environment. As a result, the fate of a mutation depends on its individual effect, epistasis with preexisting mutations and on interactions with the prevailing environment. With growing opportunities to survey dynamics of many genotypes within evolving populations, studies of both inherent properties of individual alleles and effects of their interactions in multiple conditions would address how frequently pleiotropy and epistasis guide adaptive evolution.

## Materials and Methods

### Bacterial strains and growth conditions

Twelve populations of *E. coli* have been propagated for more than 50,000 generations in Davis Minimal (DM) medium supplemented with 25 µg/ml glucose (DM25) in a long-term evolution experiment studying the dynamics and genetic basis of adaptation [43], [53]–[59]. Mutations identified in one of these populations, as described previously, are studied here [39], [57], [60]–[62]. Other types of media used in this study include Tryptic soy (Tsoy) broth, tetrazolium-arabinose (TA) agar plates, and DM media supplemented with sugars other than glucose or with glucose and additional compounds. *E. coli* strains were grown in rich Tsoy liquid media overnight from −80°C freezer stocks. Aliquots of overnight culture were transferred to 10 mL DM25 media to precondition the cultures for 24 hours prior to growth curves or fitness assays.

### Biolog phenotypic microarrays

To identify external environments that interact with the five mutations (Table 1), the respiration of the rtsgp genotype was compared to the ancestor of the long-term evolution experiment, REL606, using Biolog's Phenotypic Microarray Services in duplicate (Biolog, Hayward CA). This method utilizes a high-throughput approach to compare respiration of two strains in 1,920 different environments, consisting of a variety of carbon, nitrogen, phosphorous and sulfur sources, differences in pH, and an assortment of chemical agents that target a variety of cellular processes. This approach uses the reduction of a tetrazolium dye as a terminal electron acceptor to assess respiratory activity. The amount of respiration was quantified by the extent of color production taking readings every 15 mins and graphed as a kinetic response curve. Incubation, recording and quality control analysis of PM plates 1–20 were performed by Biolog staff using an OmniLog instrument. Relative respiration in each environment was compared using the average height of the kinetic response curves (h). The two strains were considered to have differential growth in an environment if h differed by more than 3 standard deviations of the means of h for both strains.

Since differences in respiration do not necessarily reflect differences in growth or fitness, growth rates and in some cases relative fitness (see below) of the rtsgp strain was compared with the ancestral strain in a variety of these external environments to confirm that respiration was representative of growth or fitness. These follow-up growth rate assays were confirmatory and qualitative, not quantitative (Table S2).

### Fitness assays

The fitness of each constructed strain was determined relative to the ancestor by direct competitions as described previously [53]. Briefly, competitions were typically carried out at 37°C in 10 mL of DM25, the same medium used in the original long-term evolution experiment, in 50 mL flasks with 10 mL beakers as covers. For some competitions glucose was replaced with another carbon source (β-methyl-D-glucoside) or supplemented with another compound at various concentrations (all others). These compounds and concentrations were as follows: 1.25% β-methyl-D-glucoside, 0.5 mM 3-0-β-D-galactopyranosyl-D-arabinose, 50 µM Ara-Ser, 3 mg/mL guanazole, 25 µg/ml EGTA, 100 µM Trp-Ser, 12 µg/mL piperacillin, 100 µM sodium orthovanadate, 32 µg/mL novobiocin, and 10 mM sodium nitrite. The constructed strains were competed against a marked Ara^+^ ancestral strain (REL607) that is able to utilize the sugar arabinose. The arabinose utilization phenotype was found to be neutral in each of these competitions but allowed for the two different cell types to be easily distinguishable on TA agar plates.

Competitors were pre-conditioned in the medium used for the competition for 24 hours prior to all competitions. Each competitor was then standardized based on OD600 values and added to the competition environment. Competitions were typically carried out for three days with a 1∶100 mixture transferred to fresh media every 24 hours. Since the fitness effect of some mutations was small, multiday fitness assays were used to amplify subtle advantages. Mixtures of competing strains were plated on TA agar at the start and end of each competition to determine fitness. Relative fitness (w) was calculated as the ratio of natural logarithms of realized growth by each competitor over three days of competition. Assays were typically carried out with five-fold replication and no less than three-fold replication.

The fitness values of genotypes in the selective environment assayed in our lab were generally lower than previously reported in a study carried out at the University of Houston with these strains [19]. We do not know the reason for this discrepancy, though lab-specific differences in fitness effects, for example due to differences in water source, have been seen previously [63]. We also tested whether different preconditioning methods influenced the outcome of these fitness assays (that is, preconditioning cultures in the original evolution environment (DM25) or under competition conditions). We found no significant difference in the fitness of two genotypes, tp and sgp, when competed against the ancestor under either preconditioning method (tp, *F*_2,6_ = 0.667, *P* = 0.244; sgp, *F*_2,6_ = 0.047, *P* = 0.258). Notwithstanding the difference, relative features of the fitness landscape do not seem to have changed (all five beneficial mutations remained beneficial in the selective environment (DM25) (Figure 1)). We note also that the analyses reported in this work generally consider the fitness effects of genotypes within a single environment or across two novel environments (DM25 glucose supplemented with EGTA or guanazole) used in the experiments carried out at the University of New Hampshire. Importantly, the key result observed in the dataset reported by Khan et al. [19], that epistasis was negatively correlated with expected fitness, is also seen in the work presented here.

### Fitness epistasis

Relative fitness, w, was calculated as described above based on the change in the relative density of strains in direct competition with one another. The terms that we use to describe and quantify epistasis were adopted from da Silva et al. [15]. The effect of the interactions among adaptive mutations on relative fitness was calculated as absolute epistasis:
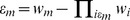
(1)where 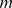 is the set of mutations, 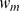 is the fitness of the genotype with the entire set of mutations, and 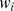 is the relative fitness of a mutant with mutation 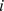 from that set. The null model assumes no interactions and under this model the fitness of a combination of beneficial mutations is equal to the product of the fitness of those mutations individually. We refer to this null hypothesis as the expected fitness of any combination of mutations. Any significant difference between the observed and expected fitness of a genotype indicates the presence of epistatic interactions. Moreover, the sign of the absolute epistasis is important, suggesting either a negative or positive interaction on the fitness of the genotype.

Genotypes consisting of more than two adaptive mutations were further analyzed for net higher-order epistatic interactions, defined as epistasis that occurs between three or more mutations that cannot be explained as the result of constituent lower-order interactions. As a result, net higher-order epistasis was calculated by subtracting the effect of lower-order interactions as shown in equation 2,
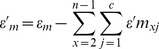
(2)where 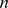 represents the number of mutations present and 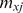 represents the fitness of a subset of the mutations present. We used this combination of methods to determine what types of interactions are most important in producing the observed phenotypes.

Given the error inherent to calculations of expected fitness and hence 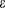, we used the method of error propagation to approximate the error of both parameters [64]. Since expected fitness of a particular genotype is equal to the product of the fitness of those mutations individually, the error (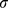) is calculated from the sum of the relative errors of the individual mutations as shown in equation 3,
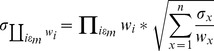
(3)where 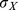 is the standard deviation of single-mutation fitnesses present. Since the uncertainty of ε depends on both 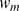 and 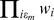, the error of 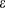 is the summation of the uncertainty of both as shown in equation 4,
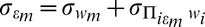
(4)Epistasis was considered significant using a *t*-test with the *t*-statistic calculated as the ratio of the mean relative fitness to its standard deviation and the degrees of freedom based on the number of replicate assays to determine significance (Table S5 and S6).

### Growth curves

To identify potential epistatic interactions among the five beneficial mutations in different environments, growth over 24 hours was quantified for the constructed strains containing only one of the five beneficial mutations and compared to both the ancestral strain and the constructed strain containing all five mutations, rtsgp. Cells were grown in 200 µL of DM25 media in 96-well plates with 12 replicates per strain. Relative growth was quantified as AUC based on OD600 measured every 15 minutes for 24 hours, compared to the ancestor, REL606, and averaged across replicates. Average relative growth of genotypes containing only a single mutation were then used to calculate an expected additive value for gp and rtsgp assuming no epistatic interactions between mutations. The error for expected values was approximated using the method of error propagation described above. Observed and expected relative growth for both gp and rtsgp was compared in each environment using a *t*-test with the *t*-statistic calculated as the ratio of the mean relative growth to its standard deviation and the degrees of freedom based on the number of replicate assays to determine significance (Table S9, S10).

## Supporting Information

Figure S1 Model II regression of observed and expected fitness under the multiplicative null model in the guanazole environment (red). The Standard Major Axis regression (SMA) was used in this analysis. Solid grey lines represent 95% confidence intervals. The dotted line signifies unity between observed and expected fitness (intercept of 0, slope of 1) and represents the null model assuming no epistasis.
(PDF)

Figure S2 Model II regression of observed and expected fitness under the multiplicative null model in the EGTA environment (red). The Standard Major Axis regression (SMA) was used in this analysis. Solid grey lines represent 95% confidence intervals. The dotted line signifies unity between observed and expected fitness (intercept of 0, slope of 1) and represents the null model assuming no epistasis.
(PDF)

Figure S3 Relationships between relative epistasis and expected fitness assuming no epistasis in the guanazole environment. Symbol color and shape defines the genotypes either containing (+, red) or lacking (−, blue) the mutation of interest defined in the top right-hand corner. ‘-interaction’ and ‘-mutation’ P-values indicate the reduction in ANCOVA model fit when the mutation×independent variable interaction or mutation main effect terms are dropped from the full model.
(PDF)

Figure S4 Relationships between relative epistasis and expected fitness assuming no epistasis in the EGTA environment. Symbol color and shape defines the genotypes either containing (+, red) or lacking (−, blue) the mutation of interest defined in the top right-hand corner. ‘-interaction’ and ‘-mutation’ P-values indicate the reduction in ANCOVA model fit when the mutation×independent variable interaction or mutation main effect terms are dropped from the full model.
(PDF)

Table S1 Biolog environments displaying differences in respiration between rtsgp and the ancestral strain.
(DOCX)

Table S2 External environments exhibiting differences in respiration and growth between ancestor and rtsgp genotypes.
(DOCX)

Table S3 Changes in fitness along each mutational trajectory in the EGTA environment.
(DOCX)

Table S4 Changes in fitness along each mutational trajectory in the guanazole environment.
(DOCX)

Table S5 Epistatic interactions in DM25+EGTA.
(DOCX)

Table S6 Epistatic interactions in DM25+guanazole
(DOCX)

Table S7 Significant interactions identified by six-way ANOVA.
(DOCX)

Table S8 Variance partitioning of interaction effects of six-way ANOVA.
(DOCX)

Table S9 Observed versus expected relative growth of the gp genotype in different external environments displaying epistatic interactions.
(DOCX)

Table S10 Observed versus expected relative growth of the rtsgp genotype in different external environments displaying epistatic interactions.
(DOCX)
