# Supplementary figures and images for: The Environment Affects Epistatic Interactions to Alter the Topology of an Empirical Fitness Landscape
Source: PLoS Genet. 2013 Apr 4;9(4):e1003426. doi: 10.1371/journal.pgen.1003426 (PMC3616912; doi:10.1371/journal.pgen.1003426)

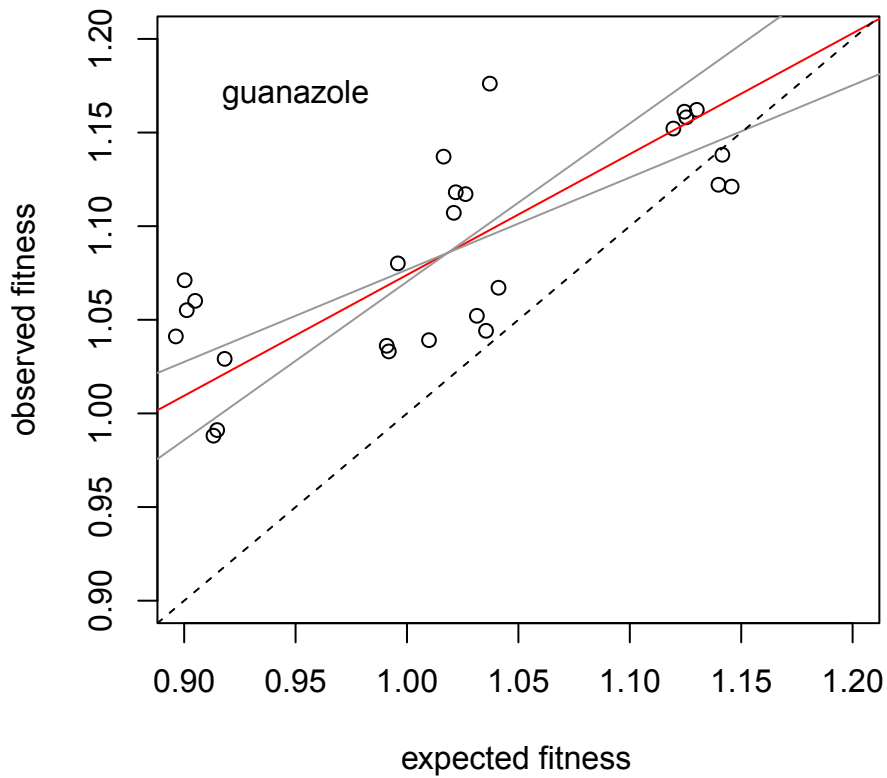

Supplement: Figure S1 — Model II regression of observed and expected fitness under the multiplicative null model in the guanazole environment (red). The Standard Major Axis regression (SMA) was used in this analysis. Solid grey lines represent 95% confidence intervals. The dotted line signifies unity between observed and expected fitness (intercept of 0, slope of 1) and represents the null model assuming no epistasis. (PDF) [file pgen.1003426.s001.pdf]

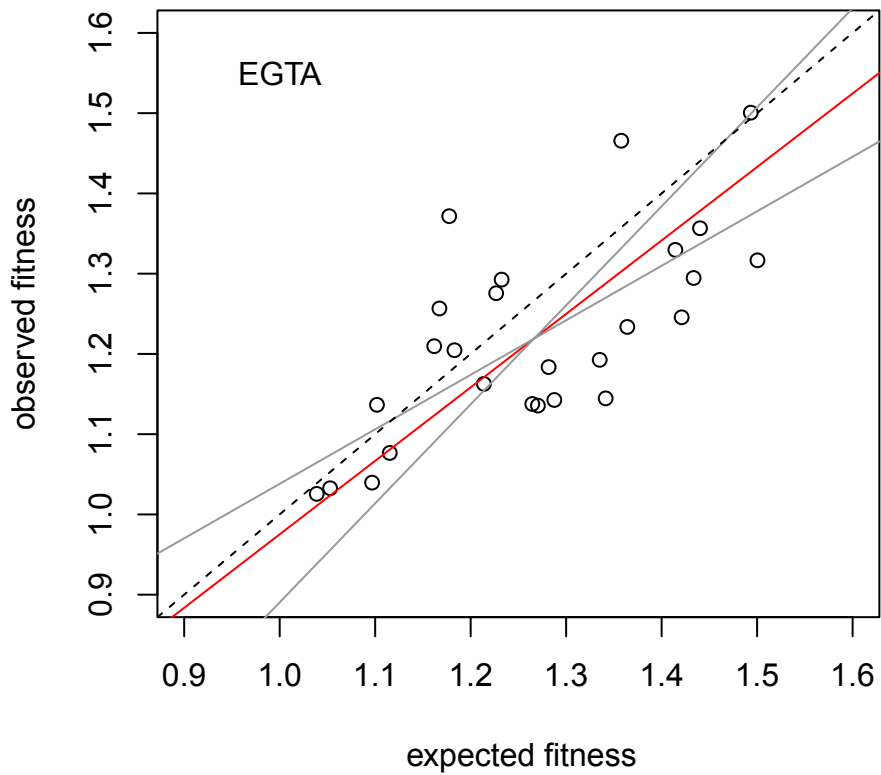

Supplement: Figure S2 — Model II regression of observed and expected fitness under the multiplicative null model in the EGTA environment (red). The Standard Major Axis regression (SMA) was used in this analysis. Solid grey lines represent 95% confidence intervals. The dotted line signifies unity between observed and expected fitness (intercept of 0, slope of 1) and represents the null model assuming no epistasis. (PDF) [file pgen.1003426.s002.pdf]

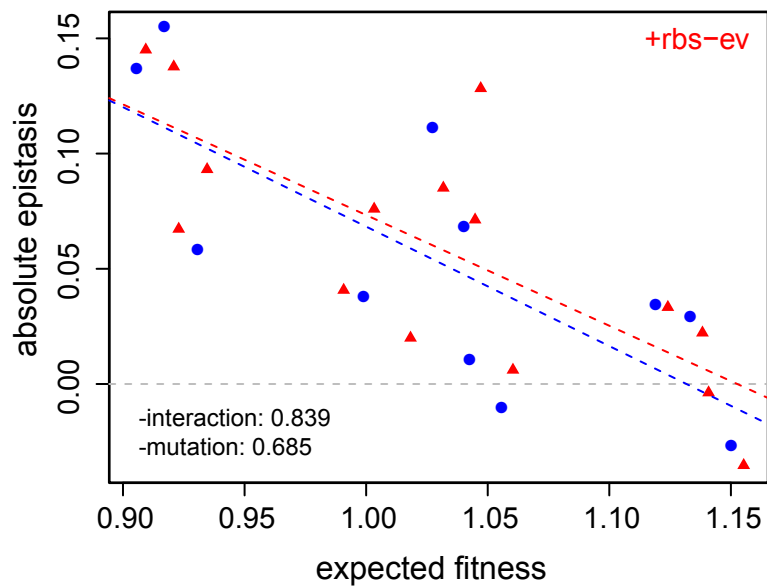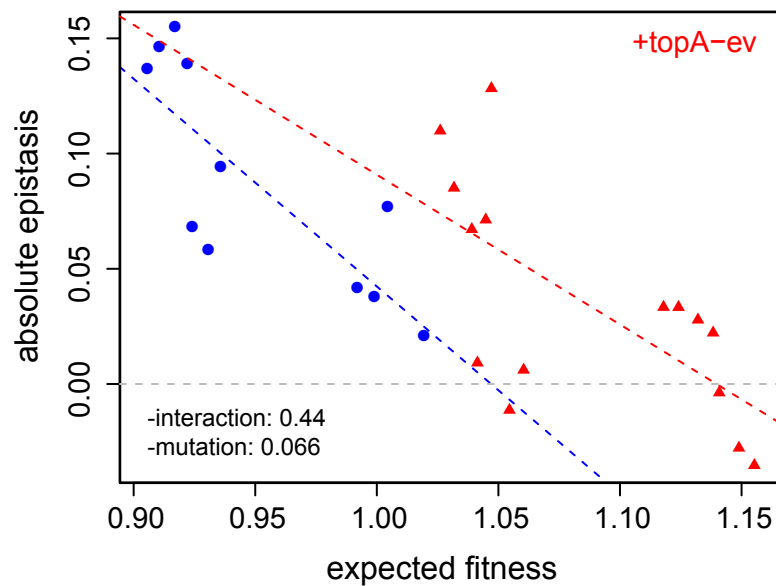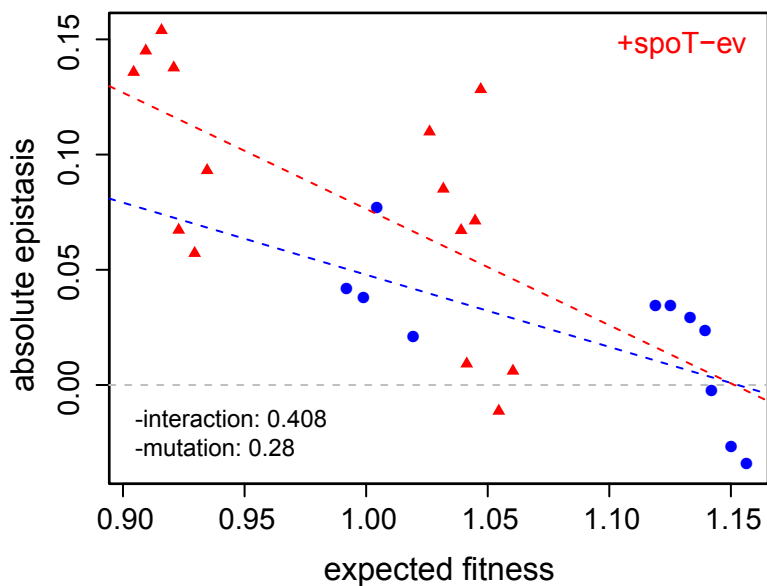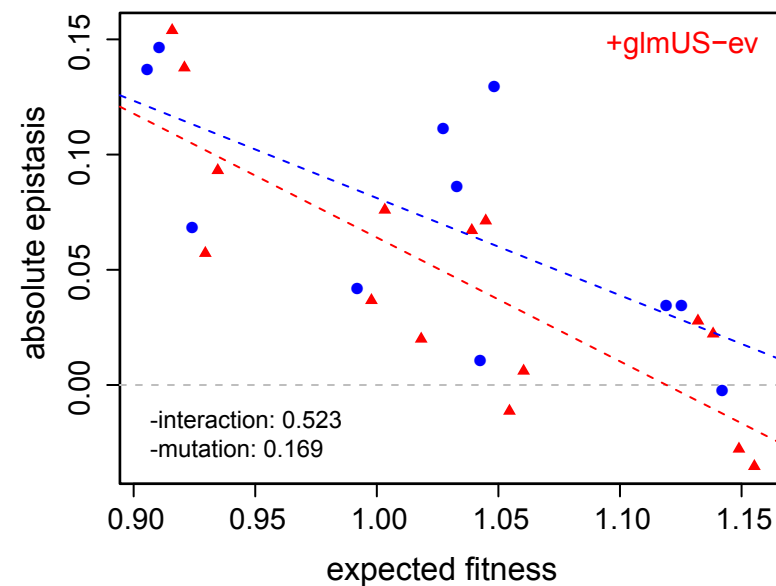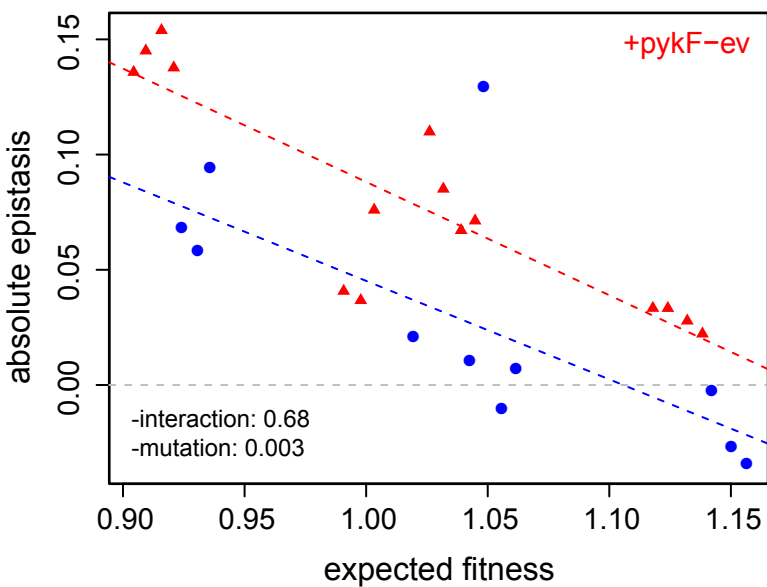

Supplement: Figure S3 — Relationships between relative epistasis and expected fitness assuming no epistasis in the guanazole environment. Symbol color and shape defines the genotypes either containing (+, red) or lacking (−, blue) the mutation of interest defined in the top right-hand corner. ‘-interaction’ and ‘-mutation’ P-values indicate the reduction in ANCOVA model fit when the mutation×independent variable interaction or mutation main effect terms are dropped from the full model. (PDF) [file pgen.1003426.s003.pdf]

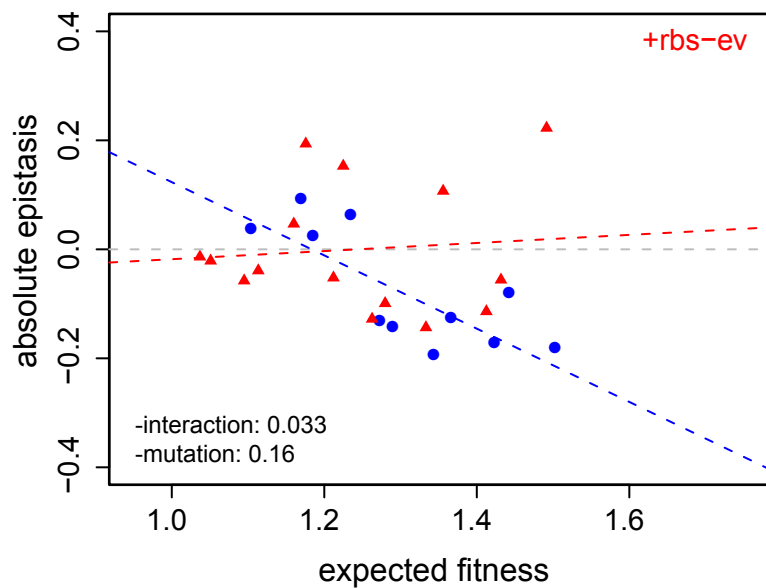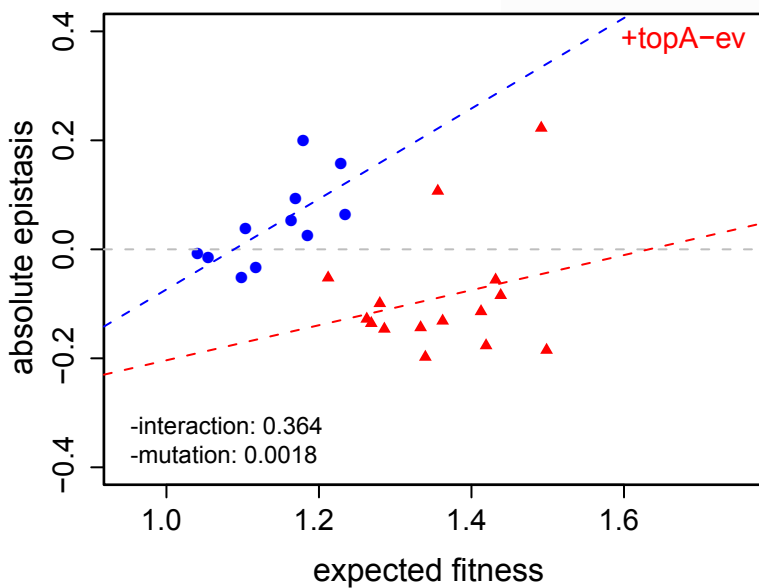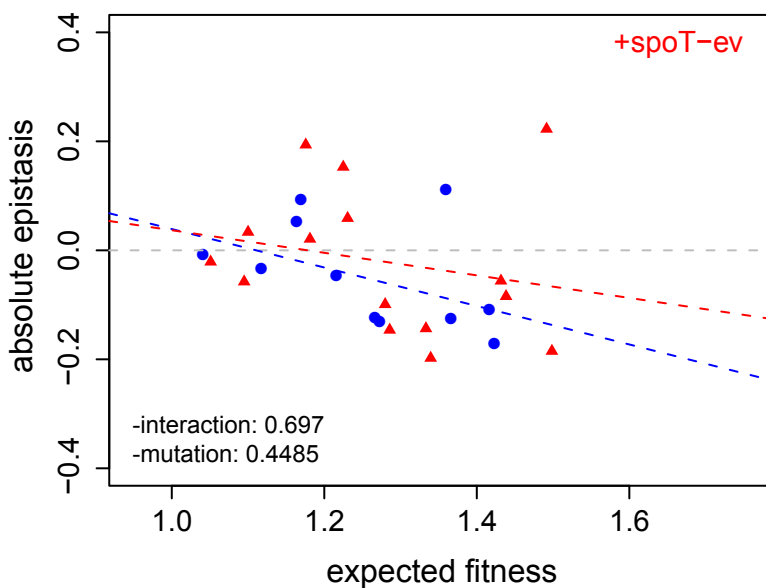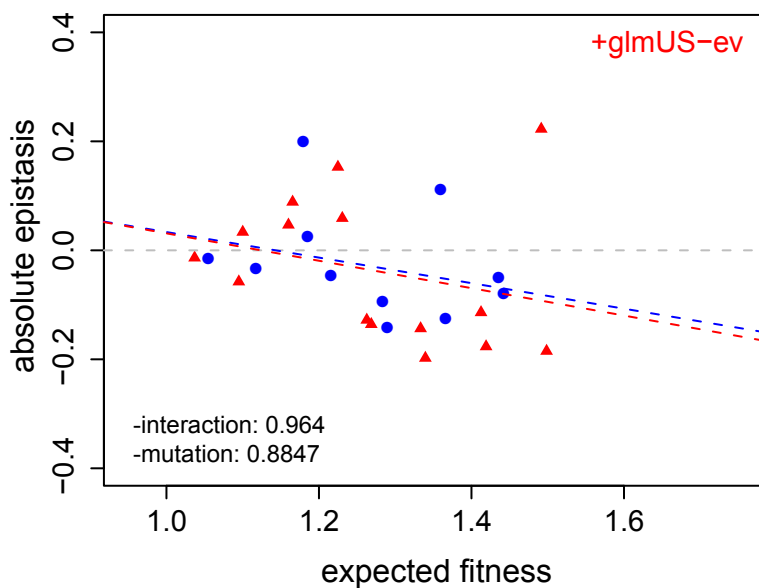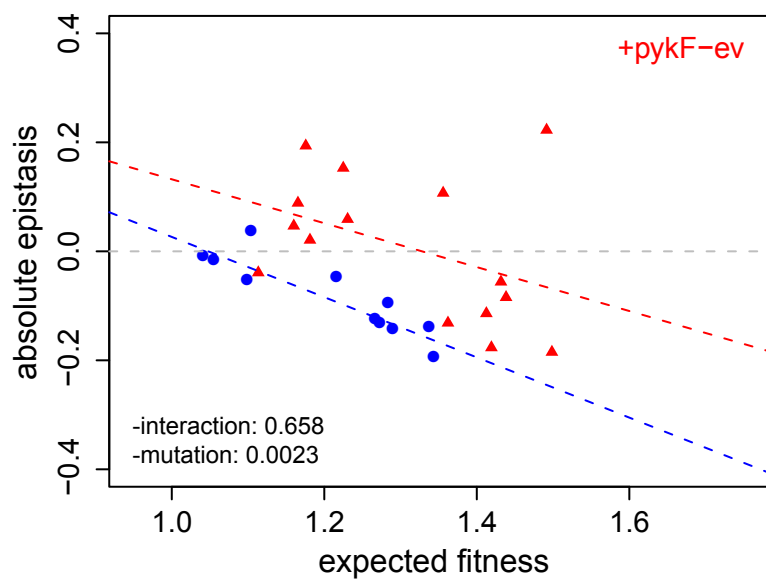

Supplement: Figure S4 — Relationships between relative epistasis and expected fitness assuming no epistasis in the EGTA environment. Symbol color and shape defines the genotypes either containing (+, red) or lacking (−, blue) the mutation of interest defined in the top right-hand corner. ‘-interaction’ and ‘-mutation’ P-values indicate the reduction in ANCOVA model fit when the mutation×independent variable interaction or mutation main effect terms are dropped from the full model. (PDF) [file pgen.1003426.s004.pdf]
