# Supplementary material for: The Environment Affects Epistatic Interactions to Alter the Topology of an Empirical Fitness Landscape
Source: PLoS Genet. 2013 Apr 4;9(4):e1003426. doi: 10.1371/journal.pgen.1003426 (PMC3616912; doi:10.1371/journal.pgen.1003426)
Supplement: Table S1 — Biolog environments displaying differences in respiration between rtsgp and the ancestral strain. (DOCX) [file pgen.1003426.s005.docx]

Table S1. Biolog Environments Displaying Differences in Respiration between rtsgp and the ancestral strain

| Biolog Plate | Well | Δh* | Biolog Plate | Well | Δh* | Biolog Plate | Well | Δh* | Biolog Plate | Well | Δh* | Biolog Plate | Well | Δh* |
| --- | --- | --- | --- | --- | --- | --- | --- | --- | --- | --- | --- | --- | --- | --- |
| PM1 | A08 | 49.5 | PM4A | G04 | -20.5 | PM9 | A02 | 16 | PM13B | D10 | 25.5 | PM17A | D02 | -28.5 |
| PM1 | G03 | 33.5 | PM5 | E02 | -15.5 | PM9 | A01 | 56 | PM13B | F11 | -36 | PM17A | E12 | 26.5 |
| PM1 | C11 | 32 | PM5 | A03 | -21.5 | PM9 | G12 | 30 | PM13B | G05 | 34.5 | PM17A | F02 | -5.5 |
| PM1 | C10 | 22 | PM5 | B04 | -13 | PM9 | H02 | 66 | PM13B | F03 | 35 | PM17A | H02 | 39.5 |
| PM1 | C07 | 43.5 | PM5 | C02 | -20 | PM9 | G01 | 26 | PM13B | A09 | 40.5 | PM18C | G07 | -59.5 |
| PM1 | F08 | 36.5 | PM5 | C03 | -20.5 | PM10 | H02 | 36 | PM13B | F08 | 33 | PM18C | F02 | -30 |
| PM1 | E08 | 74 | PM5 | C04 | -18 | PM10 | H06 | 27.5 | PM13B | D01 | 31 | PM18C | E08 | -6 |
| PM1 | B05 | 31.5 | PM5 | C05 | -15 | PM10 | H12 | 34 | PM13B | A02 | 23.5 | PM18C | D07 | 1 |
| PM1 | B11 | 37.5 | PM5 | C06 | -21.5 | PM10 | H08 | 34.5 | PM13B | B01 | 34.5 | PM18C | F09 | 16.5 |
| PM1 | A11 | 46 | PM5 | F09 | -10 | PM10 | H01 | 11.5 | PM14A | H01 | 47 | PM18C | H09 | 20 |
| PM1 | C04 | -108.5 | PM5 | D11 | -14.5 | PM10 | G11 | -26.5 | PM14A | B06 | 43 | PM18C | C09 | 25.5 |
| PM1 | F10 | 30.5 | PM6 | A06 | 33.5 | PM10 | G10 | -15.5 | PM14A | B05 | 63 | PM18C | A03 | 27.5 |
| PM1 | G07 | 23.5 | PM6 | A09 | 34 | PM10 | F10 | -6 | PM14A | A01 | 61.5 | PM18C | B05 | 32 |
| PM1 | C02 | 22 | PM6 | E01 | 50 | PM10 | F06 | 3.5 | PM14A | D07 | 9 | PM18C | B01 | 36.5 |
| PM1 | H02 | 29.5 | PM6 | E12 | 47 | PM10 | B06 | 10.5 | PM14A | F03 | -4.5 | PM18C | A09 | 38.5 |
| PM1 | E12 | 36.5 | PM6 | H01 | 12.5 | PM10 | C12 | 11 | PM14A | E01 | 42.5 | PM18C | H05 | 39 |
| PM1 | D12 | 25.5 | PM6 | H02 | 3.5 | PM10 | A05 | 37.5 | PM15B | B09 | 86.5 | PM18C | C01 | 40.5 |
| PM2A | G05 | 19.5 | PM7 | A07 | 32 | PM10 | A06 | 40 | PM15B | A01 | 45.5 | PM18C | A05 | 43.5 |
| PM2A | H11 | -8.5 | PM7 | A10 | 14.5 | PM10 | A07 | 43 | PM15B | E01 | 46 | PM18C | E01 | 45.5 |
| PM2A | C07 | 28 | PM7 | A11 | 24 | PM10 | A08 | 39.5 | PM15B | A06 | 35.5 | PM18C | G01 | 49.5 |
| PM2A | A07 | 16.5 | PM7 | B01 | 35 | PM11C | A11 | 29 | PM15B | E09 | 24.5 | PM19 | G10 | 36 |
| PM2A | A03 | 11.5 | PM7 | B11 | 16.5 | PM11C | D01 | 32.5 | PM15B | F10 | 77.5 | PM19 | A09 | 42 |
| PM2A | A05 | 21.5 | PM7 | D01 | 31.5 | PM11C | F10 | 33 | PM15B | G04 | 14 | PM19 | B01 | 24 |
| PM2A | A04 | 26.5 | PM7 | F01 | 33 | PM11C | C01 | 34 | PM15B | G09 | 32.5 | PM19 | E08 | 4.5 |
| PM2A | B06 | 9 | PM7 | G02 | 52.5 | PM11C | H09 | 34.5 | PM15B | H01 | 49 | PM19 | D09 | 41 |
| PM2A | B07 | 12.5 | PM7 | H03 | 36 | PM11C | G01 | 37 | PM15B | H06 | 43.5 | PM19 | C09 | 34 |
| PM2A | C04 | 5 | PM7 | H05 | 28.5 | PM11C | G09 | 37 | PM15B | H10 | 24 | PM19 | B09 | 44.5 |
| PM2A | E02 | 19.5 | PM8 | A04 | 26.5 | PM11C | G05 | 40 | PM15B | A10 | 35 | PM19 | E01 | 91.5 |
| PM3B | A07 | 27.5 | PM8 | A05 | 32 | PM11C | A01 | 42.5 | PM15B | B04 | 39 | PM20B | C07 | 1 |
| PM3B | H02 | 33.5 | PM8 | A06 | 18 | PM11C | E01 | 45 | PM16A | B01 | 38.5 | PM20B | A12 | 55 |
| PM3B | H06 | 30 | PM8 | G04 | -11.5 | PM11C | H05 | 45 | PM16A | B07 | 38.5 | PM20B | F01 | 21.5 |
| PM3B | H07 | 25.5 | PM8 | A12 | 46 | PM12B | E02 | 26 | PM16A | E01 | 35 | PM20B | D01 | 42 |
| PM3B | H08 | 41 | PM8 | C09 | 7 | PM12B | H12 | 14.5 | PM16A | F08 | -4.5 | PM20B | E05 | 25.5 |
| PM3B | H12 | 35 | PM8 | D01 | 36 | PM12B | F04 | -2 | PM16A | G06 | 37.5 | PM20B | A01 | 39.5 |
| PM4A | C02 | -18 | PM8 | E12 | 33.5 | PM12B | D03 | 15.5 | PM16A | G12 | 37 | PM20B | H04 | 8.5 |
| PM4A | E06 | -18 | PM8 | F01 | 43 | PM12B | C01 | 43 | PM16A | H01 | 44.5 | PM20B | A05 | 48 |
| PM4A | A10 | 23 | PM8 | H01 | 46.5 | PM12B | F01 | 53 | PM16A | A08 | 38.5 | PM20B | F05 | -22 |
| PM4A | D08 | -33 | PM8 | H04 | 15.5 | PM12B | G01 | 42 | PM17A | A05 | 34.5 | PM20B | C01 | 21 |
| PM4A | A02 | -22 | PM8 | H11 | 29.5 | PM12B | H07 | 14 | PM17A | B01 | 22.5 |  |  |  |
| PM4A | H02 | -16 | PM9 | D01 | 37.5 | PM12B | A04 | 6.5 | PM17A | C03 | 26.5 |  |  |  |

* Growth of rtsgp and REL606 in 1,920 environments was determined through the reduction of a tetrazolium dye as a terminal electron acceptor to assess respiratory activity. Δh represents the mean difference in maximum height of the respiration curve between the strains in a given environment as performed by Biolog staff (rtsgp h – REL606 h).
