# Supplementary material for: The Environment Affects Epistatic Interactions to Alter the Topology of an Empirical Fitness Landscape
Source: PLoS Genet. 2013 Apr 4;9(4):e1003426. doi: 10.1371/journal.pgen.1003426 (PMC3616912; doi:10.1371/journal.pgen.1003426)
Supplement: Table S2 — External environments exhibiting differences in respiration and growth between ancestor and rtsgp genotypes. (DOCX) [file pgen.1003426.s006.docx]

Table S2. External environments exhibiting differences in respiration and growth between ancestor and rtsgp genotypes.

| **Phenotype (Biolog #)** | **Name** | **Sign** | **Kind of Metabolism** | **Concentration** |
| --- | --- | --- | --- | --- |
| **PM01 – E08** | b-Methyl-D-Glucoside | Gain | C-Source | 1.25% |
| **PM01 – C04** | D- Ribose^1^ | Loss | C- Source | N/A |
| **PM07 – G02** | Trp-Ser | Gain | N-Source | 100 uM |
| **PM14A – H03** | EGTA | Gain | Chelator, Ca^+^/Mg^+^ | 25 µg/mL |
| **PM15B – F10** | Puromycin | Gain | Protein Synthesis | 250 µg/mL |
| **PM18C – A05, A06** | Pyrophosphate | Gain | Chelator | 250 µg/mL |
| **PM18C – G07** | Guanazole | Loss | Ribonucleotide DP reductase inhibitor | 3 mg/mL |
| **PM19 – E01, E02** | FCCP | Gain | Respiration | 250 µg/mL |

^1^ From reference [[40](#_ENREF_40)]. External environments that displayed obvious differences in respiration between rtsgp and REL606 were identified using Biolog’s Phenotypic Microarray service. The Biolog # refers to the row and column of the Biolog plate where the difference was identified. A “gain” indicates that rtsgp exhibited enhanced growth over REL606 and a “loss” indicates that rtsgp was deficient compared to REL606. Results were verified by comparing growth over 24 hours in DM25 supplemented with the appropriate conditions between rtsgp and REL606. Concentration listed is the working concentration found to differentiate growth.
