# Supplementary material for: The Environment Affects Epistatic Interactions to Alter the Topology of an Empirical Fitness Landscape
Source: PLoS Genet. 2013 Apr 4;9(4):e1003426. doi: 10.1371/journal.pgen.1003426 (PMC3616912; doi:10.1371/journal.pgen.1003426)
Supplement: Table S3 — Changes in fitness along each mutational trajectory in the EGTA environment. (DOCX) [file pgen.1003426.s007.docx]

Table S3. Changes in fitness along each mutational trajectory in the EGTA environment.

| Trajectory Number | Mutation order* | Monotonically increasing fitness | Trajectory number | Mutation order | Monotonically increasing fitness | Trajectory number | Mutation order | Monotonically increasing fitness |
| --- | --- | --- | --- | --- | --- | --- | --- | --- |
| 1 | \|rtsgp†‡ | No | 41 | t\|gprs | No | 81 | gtsrp | Yes |
| 2 | \|rtspg | No | 42 | t\|gpsr | No | 82 | gtspr | Yes |
| 3 | \|rtgsp | No | 43 | tpr\|sg | No | 83 | gtprs | Yes |
| 4 | \|rtgps | No | 44 | tpr\|gs | No | 84 | gtpsr | Yes |
| 5 | \|rtp\|sg | No | 45 | tpsrg | Yes | 85 | gs\|rtp | No |
| 6 | \|rtp\|gs | No | 46 | tps\|gr | No | 86 | gs\|rpt | No |
| 7 | \|rstgp | No | 47 | tpgrs | Yes | 87 | gstrp | Yes |
| 8 | \|rstpg | No | 48 | tpgsr | Yes | 88 | gstpr | Yes |
| 9 | \|rsgtp | No | 49 | s\|rtgp | No | 89 | gsprt | Yes |
| 10 | \|rsgpt | No | 50 | s\|rtpg | No | 90 | gsptr | Yes |
| 11 | \|rsptg | No | 51 | s\|rgtp | No | 91 | gp\|rts | No |
| 12 | \|rspgt | No | 52 | s\|rgpt | No | 92 | gp\|rst | No |
| 13 | \|rgtsp | No | 53 | s\|rptg | No | 93 | gp\|trs | No |
| 14 | \|rgtps | No | 54 | s\|rpgt | No | 94 | gp\|tsr | No |
| 15 | \|rgstp | No | 55 | strgp | Yes | 95 | gpsrt | Yes |
| 16 | \|rgspt | No | 56 | strpg | Yes | 96 | gpstr | Yes |
| 17 | \|rgpts | No | 57 | stgrp | Yes | 97 | p\|rt\|sg | No |
| 18 | \|rgpst | No | 58 | stgpr | Yes | 98 | p\|rt\|gs | No |
| 19 | \|rpt\|sg | No | 59 | stprg | Yes | 99 | p\|rstg | No |
| 20 | \|rpt\|gs | No | 60 | stp\|gr | No | 100 | p\|rsgt | No |
| 21 | \|rpstg | No | 61 | sg\|rtp | No | 101 | p\|rgts | No |
| 22 | \|rpsgt | No | 62 | sg\|rpt | No | 102 | p\|rgst | No |
| 23 | \|rpgts | No | 63 | sgtrp | Yes | 103 | ptr\|sg | No |
| 24 | \|rpgst | No | 64 | sgtpr | Yes | 104 | ptr\|gs | No |
| 25 | t\|rsgp | No | 65 | sgprt | Yes | 105 | ptsrg | Yes |
| 26 | t\|rspg | No | 66 | sgptr | Yes | 106 | pts\|gr | No |
| 27 | t\|rgsp | No | 67 | sprtg | Yes | 107 | ptgrs | Yes |
| 28 | t\|rgps | No | 68 | sprgt | Yes | 108 | ptgsr | Yes |
| 29 | t\|rp\|sg | No | 69 | sptrg | Yes | 109 | psrtg | Yes |
| 30 | t\|rp\|gs | No | 70 | spt\|gr | No | 110 | psrgt | Yes |
| 31 | t\|srgp | No | 71 | spgrt | Yes | 111 | pstrg | Yes |
| 32 | t\|srpg | No | 72 | spgtr | Yes | 112 | pst\|gr | No |
| 33 | t\|sgrp | No | 73 | g\|rtsp | No | 113 | psgrt | Yes |
| 34 | t\|sgpr | No | 74 | g\|rtps | No | 114 | psgtr | Yes |
| 35 | t\|sprg | No | 75 | g\|rstp | No | 115 | pgrts | Yes |
| 36 | t\|sp\|gr | No | 76 | g\|rspt | No | 116 | pgrst | Yes |
| 37 | t\|grsp | No | 77 | g\|rpts | No | 117 | pgtrs | Yes |
| 38 | t\|grps | No | 78 | g\|rpst | No | 118 | pgtsr | Yes |
| 39 | t\|gsrp | No | 79 | gtrsp | Yes | 119 | pgsrt | Yes |
| 40 | t\|gspr | No | 80 | gtrps | Yes | 120 | pgstr | Yes |

* Genotypes are represented as follows: *r --* Δ*rbs*; *t -- topA*; *s --* *spoT*; *g --* *glmUS*; *p--*– Δ*pykF*.

† Order in which mutations are accumulated during a mutational trajectory (left to right). For example, the mutation trajectory ‘rtsgp’ represents additions in the order: r→rt→rts→rtsg→rtsgp.

‡ The pipe symbol ‘|’ indicates a mutational step that causes fitness to decrease. For example, in the trajectory ‘|rtsgp’ adding the ‘r’ mutation to the ancestral genotype causes a decline in fitness.

The 13 mutational steps with fitness declines are: Anc→r, t→rt, t→ts, t→tg, s→rs, g→rg, p→rp, sg→rsg, gp→rgp, gp→tgp, rtp→rtsp, and rtp→rtgp. A total of 77 trajectories involve these mutational steps. The six mutational steps with significant fitness declines are: t→ts, t→tg, p→rp, sg→rsg, rtp→rtsp, and rtp→rtgp. A total of 32 trajectories involve these mutational steps
