# Supplementary material for: The Environment Affects Epistatic Interactions to Alter the Topology of an Empirical Fitness Landscape
Source: PLoS Genet. 2013 Apr 4;9(4):e1003426. doi: 10.1371/journal.pgen.1003426 (PMC3616912; doi:10.1371/journal.pgen.1003426)
Supplement: Table S4 — Changes in fitness along each mutational trajectory in the guanazole environment. (DOCX) [file pgen.1003426.s008.docx]

Table S4. Changes in fitness along each mutational trajectory in the guanazole environment.

| Trajectory Number | Mutation order* | Monotonically increasing  fitness | Trajectory number | Mutation order | Monotonically increasing  fitness | Trajectory number | Mutation order | Monotonically increasing  fitness |
| --- | --- | --- | --- | --- | --- | --- | --- | --- |
| 1 | rts\|gp†‡ | No | 41 | t\|gpr\|s | No | 81 | gt\|srp | No |
| 2 | rts\|p\|g | No | 42 | t\|gp\|sr | No | 82 | gt\|spr | No |
| 3 | rt\|g\|sp | No | 43 | tpr\|s\|g | No | 83 | gtpr\|s | No |
| 4 | rt\|gp\|s | No | 44 | tprg\|s | No | 84 | gtp\|sr | No |
| 5 | rtp\|s\|g | No | 45 | tp\|s\|r\|g | No | 85 | g\|srtp | No |
| 6 | rtpg\|s | No | 46 | tp\|s\|gr | No | 86 | g\|srpt | No |
| 7 | r\|st\|gp | No | 47 | tpgr\|s | No | 87 | g\|strp | No |
| 8 | r\|st\|p\|g | No | 48 | tpg\|sr | No | 88 | g\|stpr | No |
| 9 | r\|sgtp | No | 49 | \|srt\|gp | No | 89 | g\|sp\|rt | No |
| 10 | r\|sgpt | No | 50 | \|srt\|p\|g | No | 90 | g\|sptr | No |
| 11 | r\|sptg | No | 51 | \|srgtp | No | 91 | gprt\|s | No |
| 12 | r\|spgt | No | 52 | \|srgpt | No | 92 | gpr\|st | No |
| 13 | rgt\|sp | No | 53 | \|srpt\|g | No | 93 | gptr\|s | No |
| 14 | rgtp\|s | No | 54 | \|srpgt | No | 94 | gpt\|sr | No |
| 15 | rg\|stp | No | 55 | \|str\|gp | No | 95 | gps\|rt | No |
| 16 | rg\|spt | No | 56 | \|str\|p\|g | No | 96 | gpstr | Yes |
| 17 | rgpt\|s | No | 57 | \|st\|grp | No | 97 | \|prt\|s\|g | No |
| 18 | rgp\|st | No | 58 | \|st\|gpr | No | 98 | \|prtg\|s | No |
| 19 | rpt\|s\|g | No | 59 | \|stp\|r\|g | No | 99 | \|prst\|g | No |
| 20 | rptg\|s | No | 60 | \|stp\|gr | No | 100 | \|prsgt | No |
| 21 | rpst\|g | No | 61 | \|sgrtp | No | 101 | \|prgt\|s | No |
| 22 | rpsgt | Yes | 62 | \|sgrpt | No | 102 | \|prg\|st | No |
| 23 | rpgt\|s | No | 63 | \|sgtrp | No | 103 | \|ptr\|s\|g | No |
| 24 | rpg\|st | No | 64 | \|sgtpr | No | 104 | \|ptrg\|s | No |
| 25 | trs\|gp | No | 65 | \|sgp\|rt | No | 105 | \|pt\|s\|r\|g | No |
| 26 | trs\|p\|g | No | 66 | \|sgptr | No | 106 | \|pt\|s\|gr | No |
| 27 | tr\|g\|sp | No | 67 | \|sprt\|g | No | 107 | \|ptgr\|s | No |
| 28 | tr\|gp\|s | No | 68 | \|sprgt | No | 108 | \|ptg\|sr | No |
| 29 | trp\|s\|g | No | 69 | \|spt\|r\|g | No | 109 | \|psrt\|g | No |
| 30 | trpg\|s | No | 70 | \|spt\|gr | No | 110 | \|psrgt | No |
| 31 | t\|sr\|gp | No | 71 | \|spg\|rt | No | 111 | \|pst\|r\|g | No |
| 32 | t\|sr\|p\|g | No | 72 | \|spgtr | No | 112 | \|pst\|gr | No |
| 33 | t\|s\|grp | No | 73 | grt\|sp | No | 113 | \|psg\|rt | No |
| 34 | t\|s\|gpr | No | 74 | grtp\|s | No | 114 | \|psgtr | No |
| 35 | t\|sp\|r\|g | No | 75 | gr\|stp | No | 115 | \|pgrt\|s | No |
| 36 | t\|sp\|gr | No | 76 | gr\|spt | No | 116 | \|pgr\|st | No |
| 37 | t\|g\|r\|sp | No | 77 | grpt\|s | No | 117 | \|pgtr\|s | No |
| 38 | t\|g\|rp\|s | No | 78 | grp\|st | No | 118 | \|pgt\|sr | No |
| 39 | t\|g\|srp | No | 79 | gt\|r\|sp | No | 119 | \|pgs\|rt | No |
| 40 | t\|g\|spr | No | 80 | gt\|rp\|s | No | 120 | \|pgstr | No |

* Genotypes are represented as follows: *r --* Δ*rbs*; *t -- topA*; *s --* *spoT*; *g --* *glmUS*; *p --* Δ*pykF*.

† Order in which mutations are accumulated during a mutational trajectory (left to right). For example, the mutation trajectory ‘rtsgp’ represents additions in the order: r→rt→rts→rtsg→rtsgp.

‡ The pipe symbol ‘|’ indicates a mutational step that causes fitness to decrease. For example, in the trajectory ‘rts|gp’ adding the ‘g’ mutation to the rts genotype causes a decline in fitness.

The 23 mutational steps with fitness declines are: Anc→s, Anc→p, r→rs, t→ts, t→tg, g→sg, rt→rtg, rg→rsg, ts→tsg, tg→rtg, tg→tsg, tp→tsp, rts→rtsg, rts→rtsp, rtg→rtsg, rtp→rtsp, rgp→rsgp, tsp→rtsp, tsp→tsgp, tgp→tsgp, sgp→rsgp, rtsp→rtsgp, and rtgp→rtsgp. A total of 118 trajectories involve these mutational steps. The 3 mutational steps with statistically significant fitness declines are: t→ts, tg→tsg, and rtg→rtsg. A total of 37 trajectories involve these mutational steps
