# Supplementary material for: The Environment Affects Epistatic Interactions to Alter the Topology of an Empirical Fitness Landscape
Source: PLoS Genet. 2013 Apr 4;9(4):e1003426. doi: 10.1371/journal.pgen.1003426 (PMC3616912; doi:10.1371/journal.pgen.1003426)
Supplement: Table S5 — Epistatic interactions in DM25+EGTA. (DOCX) [file pgen.1003426.s009.docx]

Table S5. Epistatic interactions in DM25 + EGTA.

| Genotype* | Relative fitness (± 95% CI) | Epistatic deviation  (± STDEV) | t statistic | *P* |
| --- | --- | --- | --- | --- |
| *rt* | 1.160 (0.010) | -0.052 (0.015) | -7.770 | 0.001 |
| *rs* | 1.030 (0.020) | -0.021 (0.026) | -1.809 | 0.145 |
| *rg* | 1.023 (0.029) | -0.014 (0.013) | -2.219 | 0.113 |
| *rp* | 1.074 (0.021) | -0.039 (0.035) | -2.510 | 0.066 |
| *ts* | 1.140 (0.025) | -0.146 (0.025) | -13.093 | 0.0002 |
| *tg* | 1.133 (0.007) | -0.136 (0.010) | -26.504 | 0.0001 |
| *tp* | 1.231 (0.026) | -0.131 (0.034) | -8.534 | 0.001 |
| *sg* | 1.134 (0.040) | 0.034 (0.024) | 2.885 | 0.063 |
| *sp* | 1.202 (0.028) | 0.021 (0.040) | 1.151 | 0.314 |
| *gp* | 1.254 (0.031) | 0.089 (0.033) | 5.302 | 0.013 |
| *rts* | 1.181 (0.039) | -0.099 (0.028) | -8.020 | 0.001 |
| *rtg* | 1.135 (0.025) | -0.128 (0.016) | -16.367 | 0.0005 |
| *rtp* | 1.463 (0.039) | 0.107 (0.036) | 6.593 | 0.003 |
| *rsg* | 1.037 (0.004) | -0.058 (0.026) | -4.402 | 0.022 |
| *rsp* | 1.369 (0.052) | 0.193 (0.042) | 10.274 | 0.0005 |
| *rgp* | 1.207 (0.057) | 0.047 (0.035) | 2.653 | 0.077 |
| *tsg* | 1.142 (0.023) | -0.198 (0.025) | -15.626 | 0.006 |
| *tsp* | 1.354 (0.036) | -0.084 (0.041) | -4.553 | 0.010 |
| *tgp* | 1.243 (0.046) | -0.176 (0.035) | -10.165 | 0.002 |
| *sgp* | 1.290 (0.027) | 0.059 (0.041) | 2.924 | 0.061 |
| *rtsg* | 1.190 (0.034) | -0.143 (0.028) | -10.274 | 0.002 |
| *rtsp* | 1.376 (0.058) | -0.140 (0.043) | -7.250 | 0.002 |
| *rtgp* | 1.299 (0.040) | -0.086 (0.037) | -4.673 | 0.019 |
| *tsgp* | 1.314 (0.034) | -0.185 (0.042) | -8.857 | 0.003 |
| *rsgp* | 1.378 (0.043) | 0.048 (0.042) | 2.276 | 0.107 |
| *rtsgp* | 1.714 (0.053) | 0.223 (0.043) | 10.284 | 0.002 |

* Genotypes are represented as follows: *r --* Δ*rbs*; *t -- topA*; *s --* *spoT*; *g --* *glmUS*; *p--*– Δ*pykF*.
