# Supplementary material for: The Environment Affects Epistatic Interactions to Alter the Topology of an Empirical Fitness Landscape
Source: PLoS Genet. 2013 Apr 4;9(4):e1003426. doi: 10.1371/journal.pgen.1003426 (PMC3616912; doi:10.1371/journal.pgen.1003426)
Supplement: Table S6 — Epistatic interactions in DM25+guanazole (DOCX) [file pgen.1003426.s010.docx]

Table S6. Epistatic interactions in DM25 + guanazole

| Genotype* | Relative fitness (± 95% CI) | Epistatic deviation  (± STDEV) | t statistic | *P* |
| --- | --- | --- | --- | --- |
| *rt* | 1.137 (0.008) | -0.004 (0.020) | -0.332 | 0.771 |
| *rs* | 0.990 (0.031) | 0.067 (0.048) | 2.424 | 0.136 |
| *rg* | 1.038 (0.021) | 0.020 (0.028) | 1.220 | 0.347 |
| *rp* | 1.032 (0.027) | 0.041 (0.025) | 2.868 | 0.103 |
| *ts* | 1.051 (0.041) | 0.010 (0.049) | 0.342 | 0.765 |
| *tg* | 1.121 (0.016) | -0.028 (0.029) | -1.648 | 0.241 |
| *tp* | 1.151 (0.065) | 0.033 (0.026) | 2.174 | 0.162 |
| *sg* | 0.987 (0.018) | 0.058 (0.053) | 2.448 | 0.071 |
| *sp* | 1.040 (0.018) | 0.136 (0.051) | 5.953 | 0.004 |
| *gp* | 1.034 (0.022) | 0.037 (0.033) | 2.527 | 0.065 |
| *rts* | 1.175 (0.082) | 0.128 (0.050) | 4.400 | 0.048 |
| *rtg* | 1.120 (0.027) | -0.035 (0.032) | -1.910 | 0.196 |
| *rtp* | 1.157 (0.017) | 0.033 (0.029) | 1.948 | 0.191 |
| *rsg* | 1.028 (0.019) | 0.093 (0.054) | 2.992 | 0.096 |
| *rsp* | 1.054 (0.031) | 0.145 (0.053) | 4.771 | 0.041 |
| *rgp* | 1.079 (0.010) | 0.076 (0.035) | 3.715 | 0.065 |
| *tsg* | 1.043 (0.019) | -0.012 (0.055) | -0.365 | 0.750 |
| *tsp* | 1.136 (0.049) | 0.110 (0.053) | 3.578 | 0.070 |
| *tgp* | 1.160 (0.020) | 0.028 (0.036) | 1.332 | 0.314 |
| *sgp* | 1.070 (0.032) | 0.154 (0.057) | 6.075 | 0.004 |
| *rtsg* | 1.066 (0.002) | 0.006 (0.056) | 0.174 | 0.878 |
| *rtsp* | 1.117 (0.049) | 0.085 (0.055) | 2.699 | 0.114 |
| *rtgp* | 1.161 (0.045) | 0.023 (0.038) | 1.021 | 0.415 |
| *tsgp* | 1.106 (0.041) | 0.067 (0.059) | 1.972 | 0.187 |
| *rsgp* | 1.059 (0.030) | 0.138 (0.058) | 4.114 | 0.054 |
| *rtsgp* | 1.116 (0.029) | 0.071 (0.060) | 2.050 | 0.177 |

*Genotypes are represented as follows: *r --* Δ*rbs*; *t -- topA*; *s --* *spoT*; *g --* *glmUS*; *p--* Δ*pykF*.
