# Supplementary material for: The Environment Affects Epistatic Interactions to Alter the Topology of an Empirical Fitness Landscape
Source: PLoS Genet. 2013 Apr 4;9(4):e1003426. doi: 10.1371/journal.pgen.1003426 (PMC3616912; doi:10.1371/journal.pgen.1003426)
Supplement: Table S7 — Significant interactions identified by six-way ANOVA. (DOCX) [file pgen.1003426.s011.docx]

Table S7. Six way ANOVA significant interactions

| Source* | DF | MS | F Ratio | Prob > F |
| --- | --- | --- | --- | --- |
| E | 1 | 1.095615 | 759.5231 | <.0001 |
| rbs | 1 | 0.086158 | 59.7278 | <.0001 |
| E*rbs | 1 | 0.007145 | 4.9535 | 0.0272 |
| topA | 1 | 0.851282 | 590.1419 | <.0001 |
| E*topA | 1 | 0.011027 | 7.6441 | 0.0062 |
| rbs*topA | 1 | 0.02108 | 14.6133 | 0.0002 |
| E*rbs*topA | 1 | 0.043871 | 30.413 | <.0001 |
| spoT | 1 | 0.05852 | 40.5681 | <.0001 |
| E*spoT | 1 | 0.165325 | 114.6094 | <.0001 |
| rbs*spoT | 1 | 0.022483 | 15.5858 | 0.0001 |
| E*rbs*spoT | 1 | 0.011506 | 7.9767 | 0.0052 |
| topA*spoT | 1 | 0.020822 | 14.4344 | 0.0002 |
| glmS | 1 | 0.032088 | 22.2446 | <.0001 |
| E*glmS | 1 | 0.010491 | 7.2725 | 0.0076 |
| topA*glmS | 1 | 0.056393 | 39.0939 | <.0001 |
| rbs*topA*glmS | 1 | 0.011573 | 8.0227 | 0.0051 |
| E*rbs*topA*glmS | 1 | 0.012116 | 8.3995 | 0.0042 |
| E*spoT*glmS | 1 | 0.020345 | 14.1042 | 0.0002 |
| rbs*spoT*glmS | 1 | 0.004597 | 3.1866 | 0.0758 |
| E*rbs*spoT*glmS | 1 | 0.013707 | 9.5025 | 0.0023 |
| topA*spoT*glmS | 1 | 0.025388 | 17.5998 | <.0001 |
| E*topA*spoT*glmS | 1 | 0.040946 | 28.3851 | <.0001 |
| rbs*topA*spoT*glmS | 1 | 0.02202 | 15.2649 | 0.0001 |
| E*rbs*topA*spoT*glmS | 1 | 0.026103 | 18.0956 | <.0001 |
| pykF | 1 | 0.945111 | 655.1883 | <.0001 |
| E*pykF | 1 | 0.412762 | 286.1427 | <.0001 |
| rbs*pykF | 1 | 0.033256 | 23.0541 | <.0001 |
| E*rbs*pykF | 1 | 0.090672 | 62.8574 | <.0001 |
| rbs*topA*pykF | 1 | 0.005856 | 4.0594 | 0.0453 |
| E*rbs*topA*pykF | 1 | 0.011543 | 8.0017 | 0.0052 |
| spoT*pykF | 1 | 0.074783 | 51.8424 | <.0001 |
| E*spoT*pykF | 1 | 0.028775 | 19.9477 | <.0001 |
| E*rbs*spoT*pykF | 1 | 0.032865 | 22.783 | <.0001 |
| rbs*topA*spoT*pykF | 1 | 0.014804 | 10.2626 | 0.0016 |
| E*rbs*topA*spoT*pykF | 1 | 0.01129 | 7.8269 | 0.0057 |
| glmS*pykF | 1 | 0.01613 | 11.1819 | 0.001 |
| E*glmS*pykF | 1 | 0.008714 | 6.0408 | 0.0148 |
| rbs*spoT*glmS*pykF | 1 | 0.030081 | 20.8532 | <.0001 |
| E*rbs*spoT*glmS*pykF | 1 | 0.012937 | 8.9685 | 0.0031 |
| topA*spoT*glmS*pykF | 1 | 0.024913 | 17.2709 | <.0001 |
| E*topA*spoT*glmS*pykF | 1 | 0.008885 | 6.1591 | 0.0139 |
| rbs*topA*spoT*glmS*pykF | 1 | 0.036642 | 25.4018 | <.0001 |
| E*rbs*topA*spoT*glmS*pykF | 1 | 0.01453 | 10.0728 | 0.0017 |
| Error | 198 | 0.2856156 |  |  |

*Full factorial model based on six binary variables to predict relative fitness of a genotype. One variable is for the environment: EGTA or guanazole. The other five represent presence or absence of each of the five mutations.
