# Supplementary material for: The Environment Affects Epistatic Interactions to Alter the Topology of an Empirical Fitness Landscape
Source: PLoS Genet. 2013 Apr 4;9(4):e1003426. doi: 10.1371/journal.pgen.1003426 (PMC3616912; doi:10.1371/journal.pgen.1003426)
Supplement: Table S8 — Variance partitioning of interaction effects of six-way ANOVA. (DOCX) [file pgen.1003426.s012.docx]

Table S8. Variance partitioning of interaction effects of six way ANOVA.

| Model | % of variance explained* | Reduction in model |
| --- | --- | --- |
| Full | 94.80 | 0.00 |
| Only GxG, GxE | 86.70 | 8.10 |
| Only GxE | 76.40 | 18.40 |
| Only GxG | 74.54 | 20.26 |
| Main effects | 64.81 | 30.00 |
