# Supplementary material for: The Environment Affects Epistatic Interactions to Alter the Topology of an Empirical Fitness Landscape
Source: PLoS Genet. 2013 Apr 4;9(4):e1003426. doi: 10.1371/journal.pgen.1003426 (PMC3616912; doi:10.1371/journal.pgen.1003426)
Supplement: Table S9 — Observed versus expected relative growth of the gp genotype in different external environments displaying epistatic interactions. (DOCX) [file pgen.1003426.s013.docx]

Table S9. Observed versus expected relative growth of the gp genotype in different external environments displaying epistatic interactions.

| **Environment** | **Observed (STDEV)** | **Expected (STDEV)** | **t statistic** | ***P*** |
| --- | --- | --- | --- | --- |
| **β-methyl-D-glucoside** | 0.967 (0.114) | 0.776 (0.137) | -4.393 | 0.002 |
| **3-0-β-D-galactopyranosyl-D-arabinose** | 0.742 (0.170) | 1.104 (0.386) | 2.097 | 0.104 |
| **Ala-Ser** | 1.006 (0.071) | 0.693 (0.184) | -3.400 | 0.042 |
| **Trp-Ser** | 0.873 (0.120) | 0.580 (0.116) | -5.049 | 0.015 |
| **Piperacillin** | 0.407 (0.048) | 1.261 (0.164) | 10.416 | 0.002 |
| **Sodium orthovanadate** | 1.450 (0.150) | 1.234 (0.226) | -2.141 | 0.099 |
| **40ºC^±^** | 1.082 (0.114) | 0.997 (0.225) | -1.249 | 0.240 |
| **Novobiocin^±^** | 1.094 (0.160) | 1.454 (0.471) | 2.295 | 0.051 |
| **Sodium Nitrite^±^** | 0.817 (0.156) | 0.490 (0.167) | -3.921 | 0.030 |

± randomly selected environments not included in the Biolog analysis.
