# Supplementary material for: The Environment Affects Epistatic Interactions to Alter the Topology of an Empirical Fitness Landscape
Source: PLoS Genet. 2013 Apr 4;9(4):e1003426. doi: 10.1371/journal.pgen.1003426 (PMC3616912; doi:10.1371/journal.pgen.1003426)
Supplement: Table S10 — Observed versus expected relative growth of the rtsgp genotype in different external environments displaying epistatic interactions. (DOCX) [file pgen.1003426.s014.docx]

Table S10. Observed versus expected relative growth of the rtsgp genotype in different external environments displaying epistatic interactions.

| **Environment** | **Observed (STDEV)** | **Expected (STDEV)** | **t statistic** | ***P*** |
| --- | --- | --- | --- | --- |
| **β-methyl-D-glucoside** | 1.657 (0.228) | 1.442 (0.392) | -1.728 | 0.118 |
| **3-0-β-D-galactopyranosyl-D-arabinose** | 0.751 (0.127) | 2.122 (0.822) | 3.730 | 0.020 |
| **Ala-Ser** | 1.449 (0.133) | 0.698 (0.254) | -5.918 | 0.010 |
| **Trp-Ser** | 1.239 (0.094) | 0.440 (0.133) | -11.985 | 0.001 |
| **Piperacillin** | 1.050 (0.032) | 1.384 (0.379) | 1.764 | 0.176 |
| **Sodium orthovanadate** | 1.950 (0.302) | 2.000 (0.501) | 0.225 | 0.833 |
| **40ºC^±^** | 1.386 (0.152) | 1.294 (0.382) | -0.807 | 0.438 |
| **Novobiocin^±^** | 1.205 (0.085) | 3.500 (1.699) | 4.052 | 0.004 |
| **Sodium Nitrite^±^** | 1.267 (0.197) | 0.251 (0.130) | -15.631 | 0.001 |

± randomly selected environments not included in the Biolog analysis.
